# A case with mesenteric desmoid tumor after laparoscopic resection of stage I sigmoid colon cancer

**DOI:** 10.1186/s40792-019-0587-8

**Published:** 2019-02-28

**Authors:** Musashi Takada, Takashi Okuyama, Ryuji Yoshioka, Tamaki Noie, Emiko Takeshita, Shinichi Sameshima, Masatoshi Oya

**Affiliations:** Department of Surgery, Saitama Medical Center, Dokkyo Medical University, 2-1-50 Minami-Koshigaya, Koshigaya, Saitama 343-8555 Japan

**Keywords:** Intra-abdominal desmoid, Laparoscopic colectomy, Colon cancer, Surgery

## Abstract

**Background:**

Intra-abdominal desmoid tumors are rare and generally occur in some patients with familial adenomatous polyposis or secondary to an external stimulus such as surgical trauma. We report herein a case of intra-abdominal desmoid tumor in the jejunal mesentery after laparoscopic colectomy for sigmoid colon cancer.

**Case presentation:**

A 74-year-old woman underwent laparoscopic sigmoid colectomy for colon cancer with pathological stage I. Follow-up computed tomography (CT) 18 months after primary surgery showed a nodular and enhanced soft tissue density mass, 20 mm in size, in the mesentery at the left side of the abdomen. Serum carcinoembryonic antigen (CEA) and carbohydrate antigen 19-9 (CA19-9) levels were within the normal range. Fluorodeoxyglucose positron emission tomography did not suggest cancer recurrence. Another CT scan, done 1 month later, revealed that the tumor had enlarged to 25 mm in size. Although the pathological diagnosis was not obtained, we suspected recurrence of the sigmoid colon cancer and applied chemotherapy using capecitabine, oxaliplatin, and bevacizumab. After 3 cycles of chemotherapy, however, the tumor had enlarged further. Therefore, the surgical resection of the tumor was performed to determine the diagnosis and to achieve possible curative resection of the tumor. The tumor existed in the mesentery of the jejunum, 100 cm from the ligament of Treitz, and showed invasive growth. We resected 40 cm of the jejunal segment together with the tumor. Microscopically, the tumor was composed of fibroblast, myofibroblast, and infiltrating the inflammatory cell and diagnosed as desmoid tumor by immunostaining (desmin+/−, β-catenin+, CD117−, vimentin+). At 33 months after the resection of the desmoid tumor, neither the sigmoid colon cancer nor desmoid tumor has had a recurrence.

**Conclusions:**

After surgery for gastrointestinal cancer, it is difficult to differentiate between intra-abdominal desmoid tumor and recurrence. The possibility of intra-abdominal desmoid should be considered along with tumor recurrence during postoperative surveillance after resection of gastrointestinal cancer, especially when the risk of recurrence is low.

## Background

Intra-abdominal desmoid tumor develops in some patients with familial adenomatous polyposis or occurs secondary to an external stimulus such as surgical trauma [1, 2]. Intra-abdominal desmoid tumor has been reported after conventional laparotomy [3], but few cases of intra-abdominal desmoid tumor after laparoscopic surgery have been reported [4–6]. We report herein a case of intra-abdominal desmoid tumor in the mesentery of the small intestine after laparoscopic sigmoid colectomy for sigmoid colon cancer.

## Case presentation

A 74-year-old woman underwent laparoscopic sigmoid colectomy with D3 lymph node dissection for sigmoid cancer at our institution. She had no clinical and familial history of familial adenomatous polyposis. Sigmoid colon cancer (S, type 1, 40 × 38 mm, tub1, pT2, med, INFβ, ly1, v0, pN0 (0/11), pathological stage I) was pathologically diagnosed from the resected specimen, and the resection was curative. The patient was regularly followed up without adjuvant chemotherapy.

**Fig. 1:**
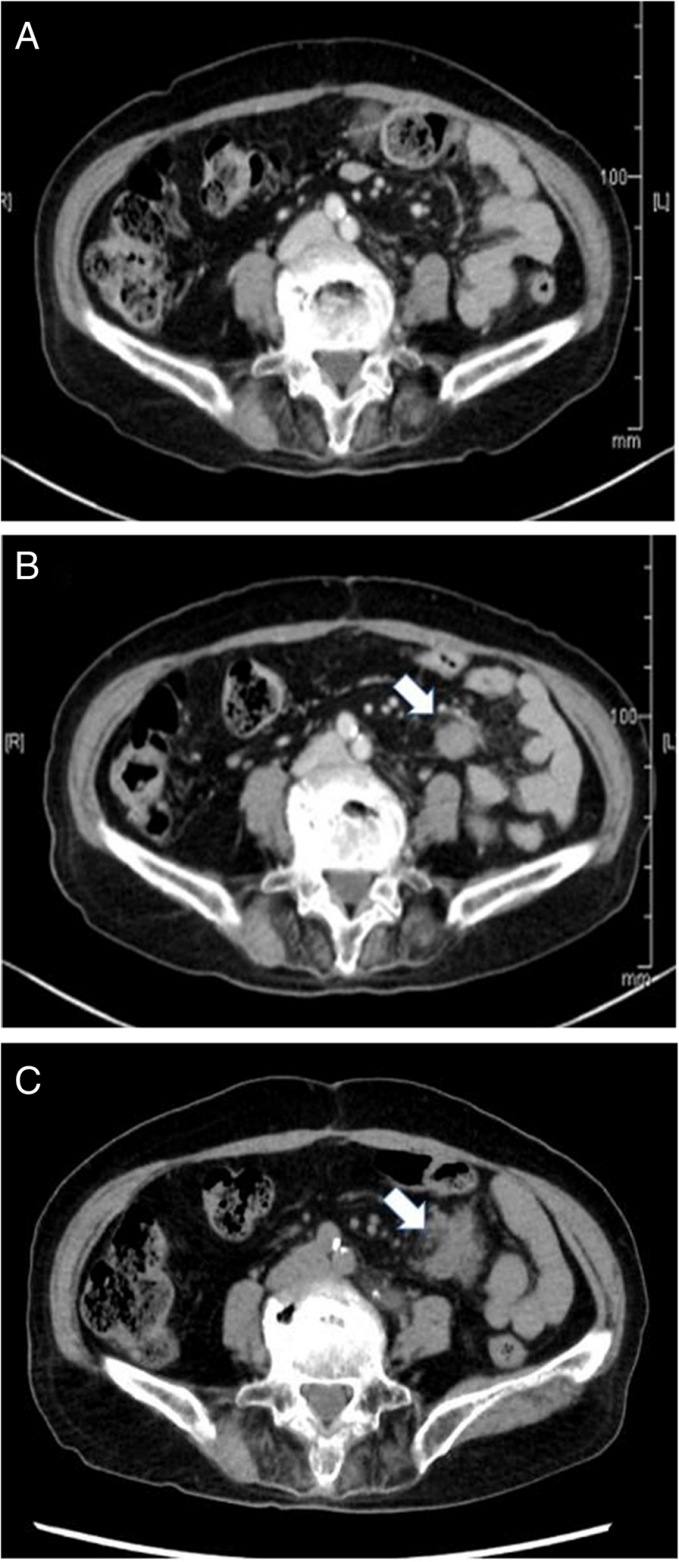
Changes in the CT findings. **a** No tumor is detected in the mesentery at 6 months after colectomy. **b** A tumor 20 mm in size was detected in the mesentery at 18 months after colectomy. CT scan showed partly unclear boundary to the surrounding tissue. **c** The tumor was enlarged up to the size of 33 mm with irregular margin after three courses of CAPOX + Bmab treatment

Eighteen months after sigmoid colectomy, a solitary abdominal tumor in the mesentery of the small intestine was detected by contrast-enhanced computed tomography (CT). The tumor was 20 mm in size, enhanced by contrast medium, and showed partly unclear boundary to adjacent tissue (Fig. 1a–c). Serum carcinoembryonic antigen (CEA) and carbohydrate antigen (CA19-9) levels were within the normal range. Although we suspected this tumor represented peritoneal recurrence, fluorodeoxyglucose positron emission tomography (FDG-PET) did not show abnormal uptake. Follow-up CT after 1 month showed that the tumor had enlarged to 25 mm in size (Fig. 1c). Although no pathological diagnosis was obtained, the radiologist of our hospital and the colorectal group conference of our department evaluated the tumor as a recurrent tumor, potentially peritoneal metastasis from the sigmoid colon cancer. We discussed about the treatment strategy for the patient in a conference and applied chemotherapy using capecitabine, oxaliplatin, and bevacizumab (CAPOX + bevacizumab (Bmab)) to observe the response of the tumor to chemotherapy and to examine whether other lesions suggestive of recurrence developed. Then, we fully explained the situation to the patient and her family. They accepted the treatment strategy we suggested.

After administration of 3 cycles of CAPOX + Bmab, the tumor showed further enlargement. At that time, we thought that histological diagnosis of the tumor was necessary before applying second-line chemotherapy. Surgical resection of the tumor was therefore performed.

**Fig. 2:**
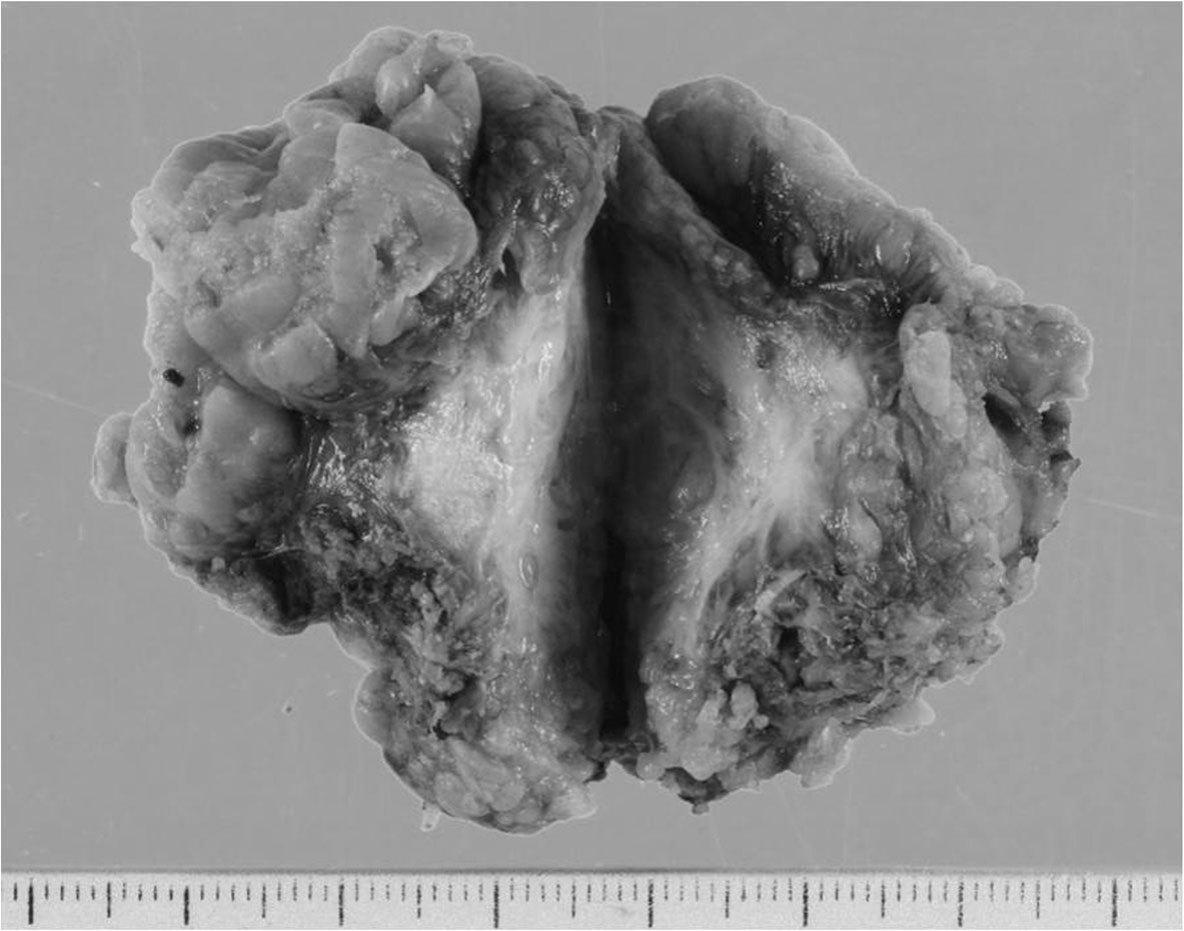
Microscopic view of the resected specimen. A tumor 31 × 21 mm in size with whitish and smooth cut surface. The margin was irregular

**Fig. 3:**
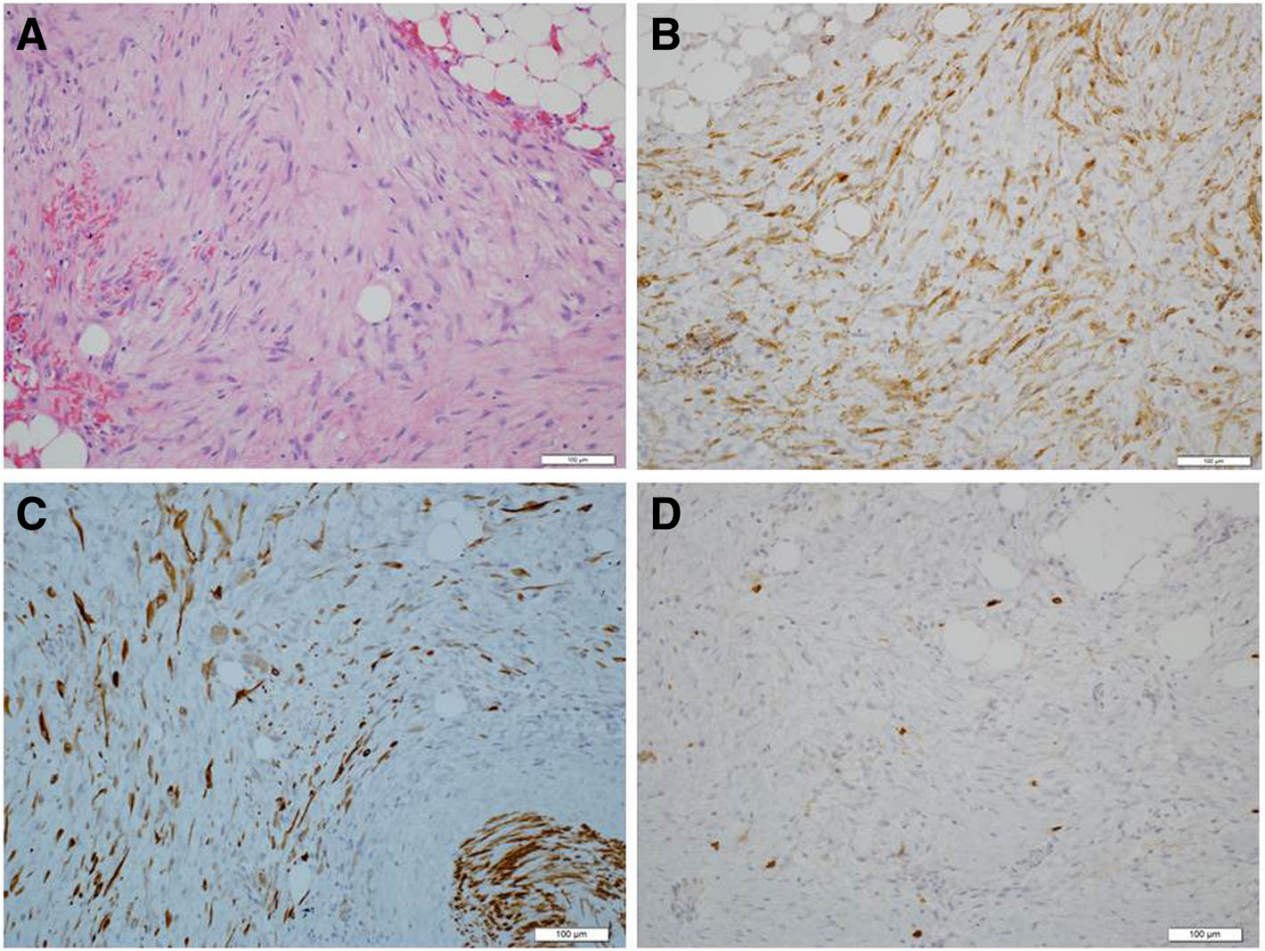
Pathological findings of the resected specimen. **a** In hematoxylin-eosin staining, the tumor consisted of relatively homogenous spindle cells with few mitoses (< 1%). **b** Both the cytoplasm and nucleus were positive in β-catenin staining. **c** In desmin staining, vascular smooth muscles were positive whereas tumor cells were marginally positive. **d** In c-kit staining, only mast cells were positive

The tumor existed in the jejunal mesentery, 100 cm from the ligament of Treitz. Although invasive growth was apparent, no evidence suggested invasion to the adjacent organs. We therefore resected 40 cm of the jejunal segment together with the tumor (Fig. 2). Microscopically, the tumor comprised fibroblasts, myofibroblasts, and infiltrating inflammatory cells. Immunohistochemical staining showed positive results for β-catenin and vimentin, focally positive results for desmin and α-smooth muscle actin (α-SMA) and S-100, and negative results for CD34 and CD117 (c-kit) (Fig. 3a–d). Based on these findings, the tumor was histologically diagnosed as a desmoid tumor. As of 36 months after resection of the desmoid tumor, neither the sigmoid colon cancer nor desmoid tumor has recurred.

## Conclusions

Desmoid tumor is a benign condition resulting from an abdominal proliferation of myofibroblasts [7]. Although well recognized as a significant complication of familial adenomatous polyposis, particularly after colectomy, desmoid tumor after colectomy for sporadic colorectal cancer is rare [8]. Since surgical trauma is thought to represent an important trigger for desmoid tumor [2], desmoid tumor after laparoscopic surgery may be even rarer than that after conventional laparotomy.

To the best of our knowledge, only nine cases have been reported previously in the literature, including case reports in Japanese (Table 1) [3, 6, 9–15]. The age and gender of patients were compatible with those of patients with colorectal cancer. These cases did not necessarily occur at sites of surgical trauma. In six of nine patients, pathological stages were stage I or II, suggesting that desmoid tumor often occurs in patients with a low risk of recurrence.

**Table 1:** Table 1

Reference No.	Author	Year	Age	Gender	Site	Size (mm)	Treatment	Preoperative diagnosis	Cancer site^b^	Stage	Interval (months)	Prognosis	Recurrence/cause of death
[7]	Kawano	2010	70	M	pre-duodenal region	30	resection	both^a^	A	II	18	6 m alive	disease free
[8]	Matagi	2011	71	M	presacral region	50	resection	recurrence	R	I	12	41 m dead	sepsis
[9]	Mizuno	2011	61	M	anastomotic site	27	resection	recurrence	A	IIIb	12	24 m alive	disease free
[10]	Gabata	2013	56	F	abdominal cavity	40	resection	non-recurrence	D	IIIa	12	11 m alive	disease free
[11]	Hamada	2014	70	M	surface of the liver	20	resection	unknown	A	0	24	12 m alive	disease free
[12]	Otani	2015	65	F	anastomotic site	40	resection	both	T	II	12	12 m alive	disease free
[13]	Saji	2015	66	M	jejunal mesenterium	24	resection	both	D	I	20	14 m alive	disease free
[14]	Kondo	2015	66	F	paraaortic region, ileocecal mesenterium	150 + 90/ 60 (two lesions) ^c^	resection	both	Ra	IIIa	12	12 m alive	disease free
[15]	Shimonozono	2018	81	M	gastrosplenic ligament	18	resection	non-recurrence	T	I	12	24 m alive	disease free
	Our case	2018	74	F	jejunal mesenterium	20	resection	recurrence	S	I	18	30 m alive	disease free

^a^both recurrence and non-recurrence were suspected

^b^A: ascending colon, R: rectum, D: descending colon, T: transverse colon, Ra: upper rectum

^c^a snowman-shape lesion 150 mm + 90 mm in size and a lesion 60 mm in size

In all patients, desmoid tumor developed within 2 years after the resection of colorectal cancer. Since patients after resection of colorectal cancer usually undergo regular follow-up, desmoid tumors were less than 50 mm in diameter in all except for one patient. All patients underwent surgical resection of the desmoid tumor, with exception of one patient who died of sepsis.

Mesenteric desmoid developing 18 months after laparoscopic surgery for a sporadic, stage I, sigmoid colon cancer, as in our patients, is rare [16–18], and we did not consider the possibility of desmoid tumor until surgical resection. Under the suspicion of peritoneal dissemination, we performed systemic chemotherapy using CAPOX + Bmab to both treat the tumor and observe the response of the tumor. During the 3 cycles of CAPOX + Bmab treatment, no recurrence tumors were identified. Moreover, tumor size was increased, suggesting a necessity for changing the chemotherapeutic regimen. Because the tumor was still resectable at that time, we determined to perform tumor resection before proceeding to chemotherapy using a second-line regimen.

Even during the resection, we did not consider the possibility of a desmoid tumor. We initially tried to perform enucleation of the tumor 50 mm in diameter and had to resect a 44-cm segment of the jejunum due to the impaired blood supply to that jejunal segment. Actually, the diagnosis of desmoid tumor was obtained by rapid pathological diagnosis during surgery. Since radical resection is the gold standard for the treatment of desmoid tumor [19], the inclusion of the jejunal segment in the resection was considered to be beneficial in terms of local recurrence.

The patient has experienced no recurrence of either colon cancer or desmoid tumor as of 36 months after resection of the desmoid tumor. Because the local recurrence rate of desmoid tumor is reportedly high [2, 19, 20], we are still conducting careful follow-up using CT at an interval of at least 6 months, particularly for detecting local recurrence of desmoid tumor.

In summary, we have reported herein a case with mesenteric desmoid tumor after laparoscopic resection of stage I sigmoid colon cancer. In case a recurrence is suspected, even if curative resection may be possible, chemotherapy can be the treatment of choice because the risk of development of subsequent further metastatic lesions is high. Although we performed systemic chemotherapy under a diagnosis of recurrence, short-term neoadjuvant chemotherapy might have limited adverse events. We concluded that the possibility of desmoid should be included among the differential diagnoses of the tumor, in particular, if an intra-abdominal mass is found during the follow-up for resection of colorectal cancer with a low risk of recurrence.

## Abbreviations

Bmab: Bevacizumab
CA19-9: Carbohydrate antigen
CAPOX: Capecitabine + oxaliplatin
CEA: Carcinoembryonic antigen
CT: Computed tomography
FDG-PET: Fluorodeoxyglucose positron emission tomography
α-SMA: α-Smooth muscle actin

## References

[1] Lefevre JH, Parc Y, Kerneis S, Goasguen N, Benis M, Parc R (2008). Risk factors for development of desmoid tumors in familial adenomatous polyposis. Br J Surg.

[2] Lopez R, Kemalyan N, Moseley HS, Dennis D, Vetto RM (1990). Problems in diagnosis and management of desmoid tumors. Am J Surg.

[3] Kawano Y, Suzuki H, Matsumoto S, Suga H, Tsuruta H, Akiya Y (2010). A case of resectable intra-abdominal desmoid tumor after resection of ascending colon cancer (in Japanese). Jpn J Gastroenterol Surg.

[4] Kaplan DB, Levine EA (1998). Desmoid tumor arising in a laparoscopic trocar site. Am Surg.

[5] Brown SB, MacDuff E, O’Dwyer PJ (2013). Abdominal wall fibromatosis associated with previous laparoscopic hernia repair. Hernia.

[6] Mizuno R, Akiyoshi T, Kuroyanagi H, Fujimoto Y, Ueno M, Oya M (2011). Intra-abdominal desmoid tumor mimicking locoregional recurrence after colectomy in a patient with sporadic colon cancer: report of a case. Surg Today.

[7] Sakorafas GH, Nissotakis C, Peros G (2007). Abdominal desmoid tumors. Surg Oncol.

[8] Vitellaro M, Sala P, Signoroni S (2014). Risk of desmoid tumors after open and laparoscopic colectomy in patients with familial adenomatous polyposis. Br J Surg.

[9] Mataki Y, Sane S, Ehi K, Arigami K, Kitazono M, Natsugoe S (2011). A case of intra-abdominal desmoid tumor occurring 1 year after low anterior resection for early rectal cancer (in Japanese). J Jpn Surg Assoc.

[10] Gabata R, Yabushita K, Takeshita M, Kobayashi R, Horikawa N, Ysukioka Y (2013). A case of intra-abdominal desmoid tumor after laparoscopic resection of descending colon cancer (in Japanese). Clin Surg.

[11] Hamada K, Nonaka T, Kitajima T, Kamahara Y, Nagata Y, Ito M (2014). A case of desmoid tumor mimicking recurrence after laparoscopic resection of ascending colon cancer (in Japanese). Nagasaki Igakkai Zasshi.

[12] Otani S, Hanayama H, Saito T, Tsuchiya T, Ito F, Miura J (2015). A case of intraabdominal desmoid tumor mimicking cancer recurrence after laparoscopic colectomy (in Japanese). Fukushima Med J.

[13] Saji M, Yamaguchi T, Matsusue R, Hata H, Otani T, Inokai I (2015). A case with desmoid tumor in the jejunal mesenterium after laparoscopic resection for a large bowel carcinoma (in Japanese). Operation.

[14] Kondo H, Yamaguchi S, Hara K, Morita Y, Suzuki A, Tashiro J (2015). A case report of intra-abdominal desmoid tumor occurring 1 year after laparoscopic low anterior resection for rectal cancer (in Japanese). J Jpn Coll Surg.

[15] Shimonozono M, Aridome K, Ando K, Aoki D, Maenohara S, Natsugoe S (2018). A case with laparoscopically resected desmoid tumor in the gastro-splenic ligament after laparoscopic transverse colectomy (in Japanese). Geka.

[16] Yang SH, Lin JK, Lai CR, Chen CC, Li AF, Liang WY (2004). Risk factors for peritoneal dissemination of colorectal cancer. J Surg Oncol.

[17] Nagata H, Ishihara S, Oba K, Tanaka T, Hata K, Kawai K, Nozawa H (2018). Development and validation of a prediction model for postoperative peritoneal metastasis after curative resection of colon cancer. Ann Surg Oncol.

[18] Mayanagi S, Kashiwabara K, Honda M, Oba K, Aoyama T, Kanda M (2018). Risk factors for peritoneal recurrence in stage II to III colon cancer. Dis Colon Rectum.

[19] Smith AJ, Lewis JJ, Merchant NB, Leung DH, Woodruff JM, Brennan MF (2000). Surgical management of intra-abdominal desmoid tumors. Br J Surg.

[20] McKinnon JG, Neifeld JP, Kay S, Parker GA, Foster WC, Lawrence W (1989). Management of desmoid tumors. Surg Gynecol Obstet.

